# China completes world's largest ground-based network for space weather monitoring

**DOI:** 10.1093/nsr/nwae354

**Published:** 2024-10-10

**Authors:** Ling Xin

**Affiliations:** science reporter based in Ohio, United States and Beijing, China

## Abstract

The network can continuously track space weather from the Sun's surface to interplanetary space and Earth's atmosphere.

Chinese scientists have completed the world's largest and most comprehensive ground-based network for monitoring the solar-terrestrial environment, aiming to improve our understanding of hazardous space weather and safeguard astronauts, spacecraft and power grids.

The 1.4-billion-yuan Chinese Meridian Project (CMP) comprises nearly 300 instruments deployed across the country and the polar regions. Working together, they can continuously track space weather from the Sun's surface to interplanetary space and Earth's atmosphere, providing a 3D view of its complex processes, and allowing faster, more accurate detection of events such as solar storms. Chi Wang, the project's chief scientist from the National Space Science Center (NSSC) of the Chinese Academy of Sciences (CAS) in Beijing, has said that the network, which took two phases and eight years to construct, is of special significance to China, with its advances in space technology.

We used to rely on data from the United States and Europe for predicting space weather, but it will no longer be the case in years to come with the implementation of the CMP project.

—Chi Wang

With China's increasing number of space missions in recent years, it has become a national priority to safeguard its fast-growing space assets, including hundreds of satellites and the crewed Tiangong space station. ‘We used to rely on data from the United States and Europe for predicting space weather, but it will no longer be the case in years to come with the implementation of the CMP project,’ Wang said. CMP's data are shared with the global scientific community, according to Wang. His team has also been pushing ahead with a more ambitious initiative, known as the International Meridian Circle Project (IMCP), to include instruments from outside China to achieve global, continuous space weather monitoring.

## END-TO-END TRACKING

The concept of space weather was first formalized in the late 1990s, Wang said. Similar to how wind, rain and other weather phenomena are caused by short-term variations in the lower atmosphere, space weather involves temporary disturbances in the space environment, typically above the middle and upper atmosphere, and are primarily caused by solar activities.

The goal of CMP Phase I was to establish a chain of 15 stations and deploy instruments such as airglow imagers, radars, lidars, ionospheric sounders and magnetometers to monitor the near-Earth space environment. Most of these stations were situated along the 120°E meridian, stretching from Mohe in the north, through Beijing and Wuhan, down to Hainan in the south and the Zhongshan Station in Antarctica [1].

The first phase, which took four years to complete (2008–2012), allowed researchers to study various layers of the near-Earth environment, including the middle and upper atmosphere, ionosphere and magnetosphere. However, it also had limitations, Wang said. The network did not encompass China's entire space environment. Many instruments had moderate resolution and could only detect large-scale structures. Also, it lacked the capability to monitor the Sun, the primary source of space weather events.

The construction of CMP Phase II (2019–2023) doubled the number of observation stations, creating networks along the 100–120°E longitudes and 30–40°N latitudes [2]. More importantly, most of the instruments deployed during this phase were independently developed by Chinese researchers. Their performance was more advanced, with some reaching world-leading levels, Wang said.

The CMP aims to address both major scientific questions and national strategic needs, he noted. Space weather involves a highly complex chain of interplanetary and geo-space responses to solar activities, and our understanding of these processes remains rather limited. Meanwhile, predicting space weather in a timely and accurate manner is crucial for various space operations, such as determining safe conditions for astronauts to conduct spacewalks or deciding when satellite operators should temporarily shut down their equipment to avoid possible damage. Collecting comprehensive space weather data can also contribute to designing more resilient spacecraft in the first place, Wang said.

## STATE-OF-THE-ART INSTRUMENTS

By integrating them into a single network, China will be able to manage them systematically and coordinate observations much more efficiently.

—Shun-Rong Zhang

Shun-Rong Zhang, a space physicist at MIT's Haystack Observatory, was impressed by the extent, variety and geographic spread of the equipment in the CMP networks. While many of the instruments have been in use in the USA and Europe for decades, they are typically managed by research teams under separate institutions or universities. ‘By integrating them into a single network, China will be able to manage them systematically and coordinate observations much more efficiently,’ he said. Their collective impact, as well as the distinctive individual facilities, will enable Chinese scientists to make major contributions to space weather monitoring and space physics research, Zhang said.

**Figure 1.**
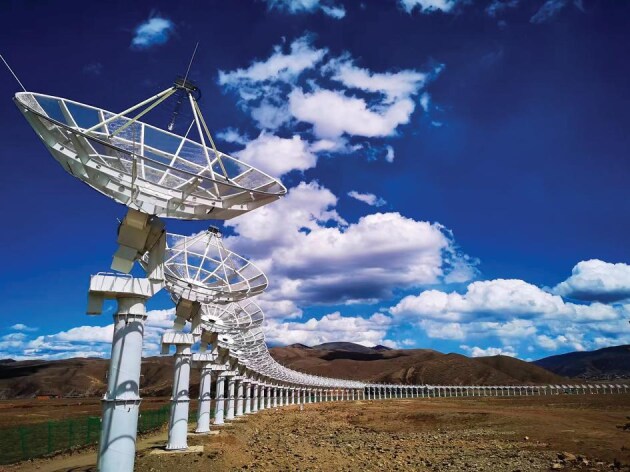
As the world's largest circular array for solar radio imaging, the Daocheng Radio Telescope (DART) consists of 313 radio antennas, each with a diameter of 6 meters. *(Courtesy of NSSC)*.

Among the cutting-edge facilities is the Daocheng Radio Telescope (DART) on the Tibetan Plateau in southwest China (Fig. 1). As the world's largest circular array for solar radio imaging, DART features a total of 313 radio antennas, each with a diameter of 6 meters, which are evenly arranged around a 3.14-kilometer-long circle [3]. It is designed to capture direct images of the dynamic solar surface with high spatial, temporal and energy resolution, said Jingye Yan, DART's chief scientist from the NSSC.

Specifically, DART works in the waveband of 150–450 megahertz to detect solar flares and coronal mass ejections. These powerful eruptions on the Sun are caused by reconnections in its magnetic field, and will release magnetized plasma in the solar upper atmosphere into interplanetary space. When the plasma reaches Earth, it may trigger geomagnetic storms that disrupt power grids, interfere with satellite operations and pose risks to astronauts aboard space stations. The team faced a number of challenges in building and operating DART, Yan said.

One major challenge was calibration, which involves aligning the dishes and ensuring accurate data syntheses. With the help of a 100-meter-tall calibration tower at the center of the array, the team was able to check the performance of each antenna, make sure they were accurately pointed, and correct any discrepancies caused by temperature changes or other factors.

DART monitors the Sun for eight hours every day. At night, it is used to study the universe, surveying the Milky Way galaxy and searching for the so-called long-period pulsars. Unlike most pulsars, which are extremely dense neutron stars spinning with periods ranging from 0.1 to a few seconds, long-period pulsars rotate much more slowly, taking anywhere from a few rotations per minute to tens of minutes per rotation. They are much harder to find, but Yan's team has already detected a few using DART.

**Figure 2.**
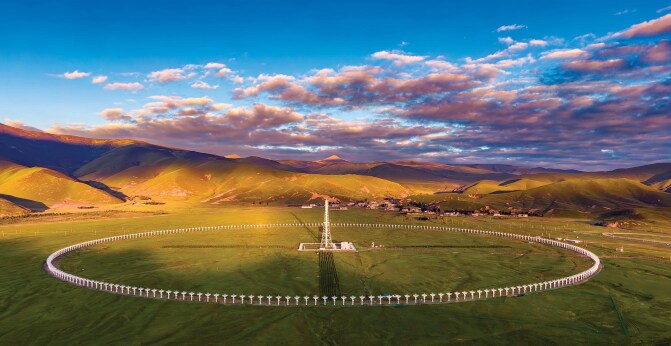
DART monitors the Sun eight hours a day. At night, it is used to study the universe, surveying the Milky Way galaxy and searching for the so-called long-period pulsars. *(Courtesy of NSSC)*.

The team hopes to share solar observation data with the international community next year. Researchers outside China will also be invited to apply for night observation time with DART, according to Yan (Fig. 2).

The Sanya ISR is an impressive installation from a technical perspective, and it was a pleasure to see that we have very similar yet complementary infrastructure.

— Axel Steuwer

Another state-of-the-art facility in the network is the Sanya Incoherent Scatter Radar Tristatic System (SYISR-TS), located on the southern island of Hainan. As one of the largest and most powerful of its kind in the world, SYISR-TS can scan Earth's ionosphere with high resolution, and detect key parameters higher than 1000 kilometers above the ground, according to Xin'an Yue, the project's chief scientist from the Institute of Geology and Geophysics (CAS) in Beijing [4].

The ionosphere, mainly located 60–1000 kilometers above, is filled with charged particles, said Xin'an Yue. By beaming high-power radio waves into this layer and having them interact with the electrons, researchers can collect and analyze the backscattered signals, and derive properties such as electron and ion densities, temperatures and ionospheric dynamics through a complicated algorithm. Since the backscattered radiation is extremely weak, the radars must be equipped with large power outputs (typically in megawatts) and extensive apertures (hundreds of square meters), making them both costly and technically challenging to build.

**Figure 3.**
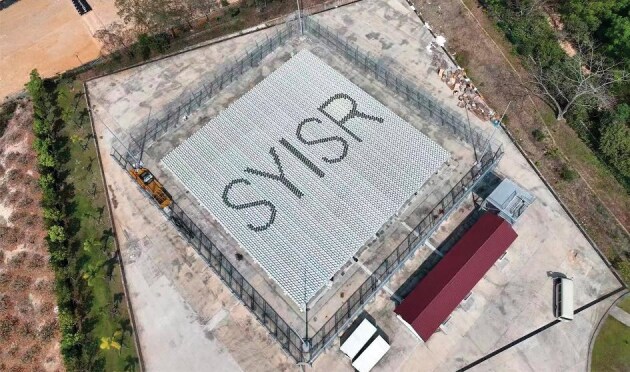
Another new facility of the CMP network is the Sanya Incoherent Scatter Radar Tristatic System, located in Hainan. As one of the largest and most powerful of its kind in the world, SYISR-TS can scan Earth's ionosphere with high resolution and detect key parameters higher than 1000 kilometers above the ground. *(Courtesy of NSSC)*.

With funding from the National Natural Science Foundation of China and CMP Phase II, Yue and his colleagues have developed a full-sized facility in Sanya in the past decade, covering 1600 square meters and comprising more than 8000 channels, along with two additional receiving stations in nearby counties over the island. ‘SYISR-TS's detection capability is similar to seeing a candle from 300 kilometers away with the naked eye,’ Yue said (Fig. 3).

Completed in 2023, the 300-million-yuan facility employs modern radar technologies to study the ionosphere's uneven structure with a resolution of dozens of meters. Sanya also makes an ideal location for SYISR-TS to study richer ionospheric processes at low latitudes. ‘The monitoring of low latitude dynamical drivers is essential for both dynamic mechanism investigations and applications,’ said Axel Steuwer, director of the European Incoherent Scatter (EISCAT) Scientific Association based in Kiruna, Sweden. ‘The Sanya Incoherent Scatter Radar Tristatic System can measure neutral wind and electric field through line-of-sight ion velocity in multiple directions simultaneously, and will significantly contribute to low latitude ionosphere dynamical and electrodynamical investigations.’

The Chinese and European teams have visited each other in Norway and Beijing, conducting fruitful discussions on possible collaboration in the future, according to Steuwer. ‘The Sanya ISR is an impressive installation from a technical perspective, and it was a pleasure to see that we have very similar yet complementary infrastructure,’ he said.

## OPPORTUNITIES AND CHALLENGES

We are far more efficient and productive when we work together

—Michel Blanc

Ground-based facilities have their limitations, and to further enhance its space weather monitoring network, China needs to extend it into space, Wang said. For instance, solar observations from Earth's surface are limited to the radio waveband. To study the Sun in more energetic bands, such as X-rays that are readily absorbed by Earth's atmosphere, telescopes need to be positioned in space.

Next year, a telescope named the Solar wind Magnetosphere Ionosphere Link Explorer (SMILE), jointly developed by CAS and the European Space Agency, is scheduled to be launched from the Guiana Space Center in Kourou [5]. Operating from a highly elliptical orbit, SMILE will investigate the interactions between the solar wind and Earth's magnetosphere, significantly advancing our understanding of the complex processes of space weather.

Following SMILE, Chinese scientists plan to develop a solar observatory known as Kuafu-2 and put it in a solar polar orbit in five years’ time. They also hope to launch a satellite constellation for multi-scale magnetosphere detection in Earth’s orbit, said Wang. Eventually, the space facilities will be combined with ground-based ones to form a 3D observation network.

Space weather events are probably more complex than previously thought. They are triggered not only by solar activities, but also by impacts from the geosystem such as earthquakes, tornadoes, volcanic eruptions and even climate change. ‘There has been a major shift in the notion of space weather to include disturbances from both above and below, as they are equally important for interpreting space weather correctly,’ said Michel Blanc, a planetary scientist from the Research Institute in Astrophysics and Planetology in Toulouse, France. As a global, systematic issue in scientific research, space weather requires collaborative efforts from countries around the world, Zhang said.

To address this, Wang, Zhang, Blanc and several other scientists have been advocating for the IMCP. The IMCP aims to expand the CMP beyond China, and integrate thousands of additional ground-based instruments, in order to form two great meridian circles and achieve all-time, all-weather monitoring.

**Figure 4.**
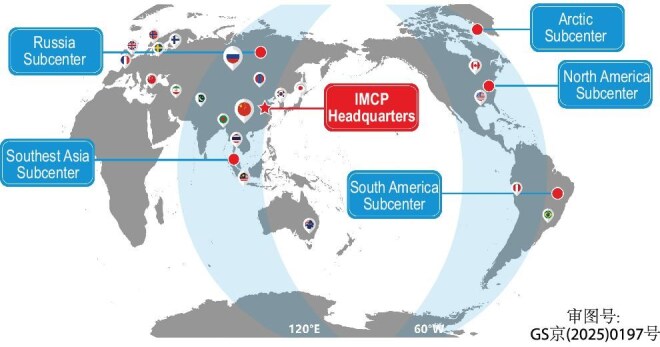
The International Meridian Circle Program aims to expand the CMP network beyond China, integrating thousands of ground-based instruments to form two great meridian circles and achieve all-time, all-weather monitoring. *(Courtesy of NSSC)*.

The primary meridian circle will be aligned along 120°E–60°W and involve observation instruments in Asia, Australia and the Americas. This circle will capture various hazards at all latitudes and facilitate the study of the effects of earthquake activities along the circum-Pacific Ring of Fire. The second IMCP circle will be along the 30°E–150°W longitudes connecting Europe, Africa and the Central Pacific. The circle can capture the residual longitude variations generated by land–ocean contrasts, particularly those related to tropospheric weather and thunderstorm activities (Fig. 4).

So far, researchers from 17 countries and regions including Brazil, Thailand, the USA, Canada, France and Russia have joined the IMCP, either in a formal capacity or as individual participants. The successful construction of the CMP has laid a solid foundation for the IMCP. With support from the Chinese Ministry of Science and Technology, Beijing municipal government and CAS, the IMCP headquarters in suburban Beijing has been established, and is ready to host representatives and researchers from participating institutions of the program.

In September, the 2024 IMCP Workshop was successfully co-hosted by the NSSC and Brazil's National Institute for Space Research in São Paulo. More than 70 scientists from countries including China, Brazil, the USA, France, South Africa, Chile, Peru, Thailand, Argentina and Paraguay attended the week-long event. They discussed a range of scientific topics, from the challenges in understanding weather processes driven by solar storms, to the South Atlantic Anomaly's effects on near-Earth space, as well as international, regional and national space weather programs. ‘We are far more efficient and productive when we work together,’ said Blanc.

***Conflict of interest statement*.** None declared.

## Author Biography

Ling Xin (辛玲) is a science reporter based in Ohio, United States and Beijing, China E-mails: lxin2015@yahoo.com; lxin.pw@gmail.com
